# Increased Reactive Oxygen Species and Cell Cycle Defects Contribute to Anemia in the RASA3 Mutant Mouse Model s*cat*

**DOI:** 10.3389/fphys.2018.00689

**Published:** 2018-06-05

**Authors:** Emily S. Hartman, Elena C. Brindley, Julien Papoin, Steven L. Ciciotte, Yue Zhao, Luanne L. Peters, Lionel Blanc

**Affiliations:** ^1^Laboratory of Developmental Erythropoiesis, Center for Autoimmune, Musculoskeletal and Hematopoietic Diseases, The Feinstein Institute for Medical Research, Manhasset, NY, United States; ^2^Donald and Barbara Zucker School of Medicine at Hofstra/Northwell, Hempstead, NY, United States; ^3^The Jackson Laboratory, Bar Harbor, ME, United States; ^4^Department of Molecular Medicine and Pediatrics, Donald and Barbara Zucker School of Medicine at Hofstra/Northwell, Hempstead, NY, United States

**Keywords:** erythropoiesis, mouse models, anemia, aplastic, bone marrow failure syndromes, reactive oxygen species (ROS), cell cycle, apoptosis

## Abstract

RASA3 is a Ras GTPase activating protein that plays a critical role in blood formation. The autosomal recessive mouse model *scat* (severe combined anemia and thrombocytopenia) carries a missense mutation in *Rasa3*. Homozygotes present with a phenotype characteristic of bone marrow failure that is accompanied by alternating episodes of crisis and remission. The mechanism leading to impaired erythropoiesis and peripheral cell destruction as evidenced by membrane fragmentation in *scat* is unclear, although we previously reported that the mislocalization of RASA3 to the cytosol of reticulocytes and mature red cells plays a role in the disease. In this study, we further characterized the bone marrow failure in *scat* and found that RASA3 plays a central role in cell cycle progression and maintenance of reactive oxygen species (ROS) levels during terminal erythroid differentiation, without inducing apoptosis of the precursors. In *scat* mice undergoing crises, there is a consistent pattern of an increased proportion of cells in the G_0_/G_1_ phase at the basophilic and polychromatophilic stages of erythroid differentiation, suggesting that RASA3 is involved in the G_1_ checkpoint. However, this increase in G_1_ is transient, and either resolves or becomes indiscernible by the orthochromatic stage. In addition, while ROS levels are normal early in erythropoiesis, there is accumulation of superoxide levels at the reticulocyte stage (DHE increased 40% in *scat; p* = 0.02) even though mitochondria, a potential source for ROS, are eliminated normally. Surprisingly, apoptosis is significantly decreased in the *scat* bone marrow at the proerythroblastic (15.3%; *p* = 0.004), polychromatophilic (8.5%; *p* = 0.01), and orthochromatic (4.2%; *p* = 0.02) stages. Together, these data indicate that ROS accumulation at the reticulocyte stage, without apoptosis, contributes to the membrane fragmentation observed in *scat*. Finally, the cell cycle defect and increased levels of ROS suggest that *scat* is a model of bone marrow failure with characteristics of aplastic anemia.

## Introduction

Every day, a healthy individual’s bone marrow produces 200 billion erythrocytes in the process of erythropoiesis. Bone marrow failure syndromes (BMFS) are a diverse group of inherited or acquired disorders characterized by varying degrees of hematopoietic failure and a predisposition to hematologic malignancies (Shimamura and Alter, 2010). While targetable molecular markers associated with the various subclasses of BMFS have recently been identified, the full molecular pathogeneses of these diseases are likely a complex interaction between genetic changes at the hematopoietic stem cell level and alterations in the hematopoietic niche of the bone marrow itself, and these mechanisms have yet to be fully elucidated (Rankin et al., 2015). Aplastic anemia (AA) is one of the most commonly diagnosed BMFS, with over 2,500 new cases each year in North America and in Europe alone, and is characterized by a hypocellular bone marrow and ineffective hematopoiesis of the erythroid, megakaryocyte, and granulocyte/monocyte lineages (Guinan, 2011; Bodine and Berliner, 2014). At the molecular level, AA is characterized by increased levels of reactive oxygen species (ROS) and defective DNA repair mechanisms (Khincha and Savage, 2013; Richardson et al., 2015).

Like most BMFS, AA can either be acquired or inherited. Acquired AA appears to be an immune-mediated suppression of the bone marrow and is thus responsive, in most cases, to immunosuppressive therapy (Miano and Dufour, 2015). Inherited AA, however, has a complex variety of pathogeneses. Although many of the patients with BMFS have identifiable causes, 30–40% of cases still have unknown etiologies, including cases of inherited AA (Peters et al., 2013). Spontaneous and engineered models of bone marrow failure have allowed focused study of these uncharacterized molecular mechanisms. One such spontaneous model is the autosomal recessive *scat* (severe combined anemia and thrombocytopenia) mouse model; *scat* carries a missense mutation in the protein-coding *Rasa3* gene (Blanc et al., 2012).

RASA3, a Ras-GTPase Activating Protein (GAP), has previously been found to play a key role in normal blood formation. This protein acts to negatively regulate the small GTPase Ras, and its localization to the membrane is required for normal function (Fukuda and Mikoshiba, 1996). The specific *Rasa3* mutation in the *scat* mouse results in the mislocalization of RASA3 to the cytosol and thus loss of RASA3 function, which leads to increased levels of active GTP-bound Ras (Blanc et al., 2012). *scat* mice interestingly cycle between hematologic crisis and remission, regardless of the cytosolic localization of RASA3, suggesting that a secreted factor may mediate the crisis-remission transition. The first crisis begins *in utero* and lasts until ∼P9. Mice that survive the first crisis will progress to remission, during which there is a striking normalization of hematologic parameters and physical appearance. Some mice then enter a second crisis, during which there is over 90% mortality by 4 weeks of age, likely due to bone marrow failure and the resulting peripheral pancytopenia (Blanc et al., 2012). Overall, the mechanism of the cyclic phenotype in *scat* mouse is unknown and offers unique opportunities to study the onset and resolution of bone marrow failure.

In this study, we further characterized the bone marrow failure in *scat* and found that RASA3 plays a central role in cell cycle progression and maintenance of ROS levels during terminal erythroid differentiation. We observed that in mice undergoing crisis episodes, the G_0_/G_1_ phase is transiently increased at the basophilic and polychromatophilic stages, suggesting that RASA3 is involved in regulating the G_1_ checkpoint. In addition, while ROS levels are normal early in erythropoiesis, we observed an accumulation of ROS at the reticulocyte stage in *scat* mice. However, mitochondria, a potential source of ROS, are eliminated normally at the reticulocyte stage, suggesting that mitochondrial metabolism and ROS production may be altered prior to removal. Surprisingly, apoptosis is not increased, but rather significantly decreased at several stages of erythroid differentiation in *scat* during crisis events. Together, these data suggest that *scat* is a model of bone marrow failure with some of the molecular characteristics of AA. To begin testing the hypothesis that a secreted factor may be mediating the cyclic phenotype, we also characterized the differences in the plasma cytokine profile of *scat* mice during crisis compared to wild type. We observed that galectin-1 is significantly decreased in *scat* mice in crisis compared to wild type.

## Materials and Methods

### Animals

All experimental mice were 14–21 days old, which corresponds to the second crisis event of the *scat* mice. All protocols were performed according to NIH animal care guidelines, as approved and enforced by the Institutional Animal Care and Use Committees at Northwell Health and The Jackson Laboratory.

### Erythroblast, Red Cell, and Plasma/Serum Collection

Bone marrow was flushed and dissociated with cold PBS containing 0.5% (weight/volume) bovine serum albumin (BSA) and 2 mM Ethylenediaminetetraacetic acid (EDTA), pH 8.0 (Invitrogen). Spleens were dissociated in cold PBS with 0.5% BSA only. Cells were filtered using a 70 μm cell strainer and CD45 depleted using anti-mouse CD45-conjugated microbeads and magnetic columns as per manufacturer instructions (Miltenyi). Circulating red cells, containing both reticulocytes and erythrocytes, were obtained by cardiac puncture. To obtain plasma or serum, whole blood with or without EDTA was centrifuged at 600 *g* for 5 min and the plasma/serum removed.

### Erythroid Surface Markers Used for Flow Cytometry

Terminal erythropoiesis was monitored using CD44, Ter119, and FSC (Forward Scatter) as markers of differentiation. While Ter119 is increased during differentiation, CD44 is lost as the erythroblasts differentiate.

Depending on the experimental purpose, reticulocyte maturation was monitored using whether CD71 and Ter119 (ROS content); or MitoTracker Red (MTR) and thiazole orange (TO, mitochondria analysis). All of these markers except for Ter119 are lost during reticulocyte maturation.

### Reactive Oxygen Species Detection by Flow Cytometry

Samples were blocked with rat anti-mouse CD16/32 (2.5 μg/10^6^ cells) for 5 min at room temperature (RT) followed by staining with a cell surface marker cocktail for 15 min at RT protected from light. Spleen and bone marrow samples were stained with rat anti-mouse CD44 APC-conjugated (0.5 μg/10^6^ cells), rat anti-mouse CD45R/CDllb/Ly6G APC-Cy7-conjugated (0.3 μg/10^6^ cells), and rat anti-mouse Ter119 V450-conjugated (0.5 μg/10^6^ cells) for CM-H2DCFDA (DCF, Invitrogen) or rat anti-mouse Ter119 FITC-conjugated (1 μg/10^6^ cells) for Dihydroethidium (DHE, Invitrogen). Peripheral blood samples were stained with rat anti-mouse Ter119 V450-conjugated (0.5 μg/10^6^ cells) and either rat anti-mouse CD71 PE-conjugated (0.2 μg/10^6^ cells) for DCF or rat anti-mouse CD71 FITC-conjugated (0.625 μg/10^6^ cells) for DHE. Samples were washed twice in PBS and stained with either DHE or DCF. DHE diluted in warm IMDM/1% FBS was added to spleen and bone marrow samples at a 0.4 μM final concentration, and to RBC at a 4 μM final concentration. DCF diluted in warm 1x PBS was added at a 25 μM final concentration for all samples. DHE and DCF were added to the samples and incubated for 30 min at 37°C protected from light_._ All samples were washed and resuspended for flow analysis. Data was collected using the BD LSRFortessa cytometer and subsequently analyzed with FlowJo software. All cell surface marker antibodies were from BD Biosciences.

### Cell Cycle Analysis by Flow Cytometry

Bone marrow cells were isolated and CD45-depleted as above, blocked with rat anti-mouse CD16/32 (2.5 μg/10^6^ cells) for 5 min at RT, and stained with an antibody cocktail of rat anti-mouse Ter119 FITC-conjugated (1 μg/10^6^ cells), CD44 APC-conjugated (0.5 μg/10^6^ cells), and CD45R/CDllb/Ly6G APC-Cy7-conjugated (0.3 μg/10^6^ cells) for 15 min at RT in the dark (Liu et al., 2013). Samples were washed in PBS/0.5% BSA, resuspended in 10 μg/mL Hoechst 33342 (Invitrogen) in warm IMDM/1%FBS (1 mL/10^6^ cells), and incubated at 37°C for 30 min in the dark. Samples were resuspended in 500 μL of 1× PBS with the addition of 0.25 μg of 7AAD (BD Biosciences) to identify dead cells. Data was collected using the BD LSRFortessa cytometer and analyzed using FlowJo software.

### Cytokine Array

Plasma was separated from circulating red cells as described above and then frozen at -80°C until future use. Cytokine levels were semi-quantitated using the Abcam 97-target Mouse Cytokine Antibody Array according to the manufacturer’s protocol (ab169820). Briefly, plasma samples were diluted 1:20 in the 1× blocking buffer provided. Membranes were imaged with the Bio-Rad ChemiDoc^TM^ MP. Quantification was performed using Gilles Carpentier’s *Dot Blot Analyzer for ImageJ* from the ImageJ website macros/toolsets folder, according to documentation at image.bio.methods.free.fr/dotblot.html.

### ELISA Analysis of Galectin-1 Levels in Mouse Plasma and Serum

Plasma samples were obtained as mentioned above, and serum samples were obtained in the same manner but without the use of EDTA. Both plasma and serum samples were used to demonstrate consistency across collection methods (de Jager et al., 2009). Prior to loading, all samples were diluted 1:10 in dilution buffer provided with Abcam’s Mouse Galectin 1 ELISA Kit (ab119595), and the kit was run according to manufacturer’s protocol. The plate was read on a spectrophotometer at 450 nm at RT. Galectin-1 concentrations of each sample were extrapolated using the standard curve created on Microsoft Excel and analyzed by comparing *scat* and wild type measures by unpaired *t*-test using GraphPad Prism software.

### Apoptosis Analysis by Flow Cytometry

Apoptosis was assessed using Annexin V as per manufacturer (BD Biosciences). All samples were stained with the cell surface marker antibodies as discussed in the cell cycle methods section, followed by staining with Annexin V and 7AAD. The protocol was modified by using 1 μg of Annexin V and 0.25 μg of 7AAD for 1 × 10^6^ cells. Apoptosis data was collected using the BD LSRFortessa cytometer and analyzed using FlowJo software.

### Mitochondria Analysis

Mitophagy was assessed using MitoTracker Red CMXRos (MTR; Molecular Probes) and TO (BD Biosciences) as described (Zhang et al., 2009). Doubly stained reticulocytes were obtained by sequential staining with MTR, then TO. MTR fluorescence emission between 600 and 620 nm was collected after 562 nm laser excitation using a BD LSR II flow cytometry analyzer (BD Biosciences).

### Transmission Electron Microscopy

For analysis by transmission electron microscopy, peripheral blood reticulocytes and red cells were fixed in 4% paraformaldehyde, 0.1% glutaraldehyde, and 1% sucrose in 0.1 M cacodylate buffer for 1 h at 4°C, then washed in 0.1 M buffer at pH 7.4; remaining aldehydes were quenched with 50 mM ammonium chloride. The fixed cells were then dehydrated and embedded in LR-White Resin, sectioned to 50–70 nm thickness, and examined in a Philips-410 electron microscope.

### Statistics

Statistical evaluations between different experimental groups were performed using GraphPad Prism 7 (unpaired *t*-test) and *p < 0.05* was considered to indicate statistical significance.

## Results

### *scat* Presents With Cell Cycle Defects at the Basophilic and Polychromatophilic Stages

**FIGURE 1:**
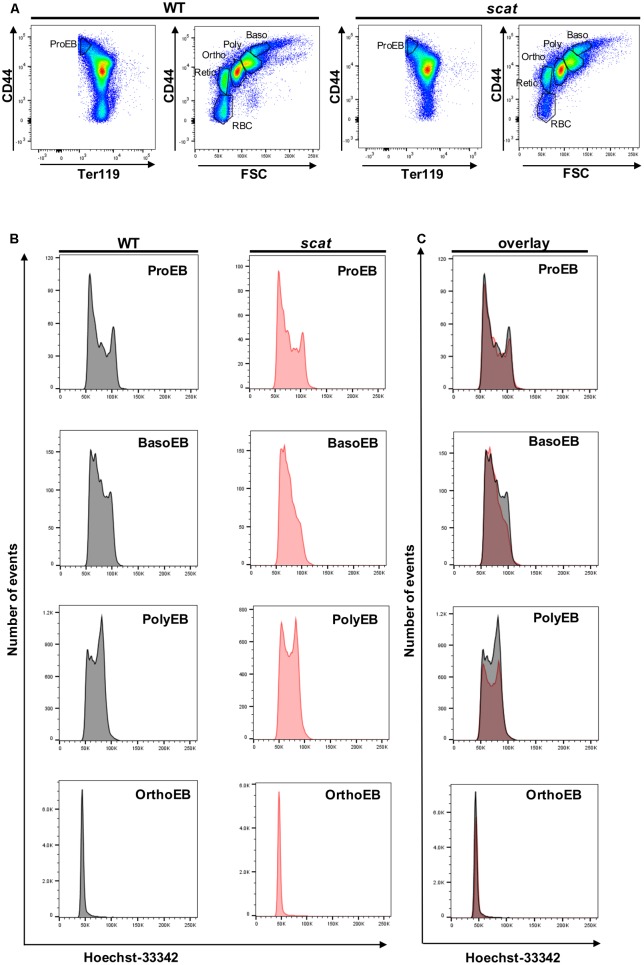
Hoechst staining reveals altered cell cycling in the basophilic and polychromatophilic stages of erythropoiesis in *scat* mice. **(A)** Gating of CD45-depleted, Ter119 positive bone marrow cells based on CD44 surface expression in wild type and *scat*. **(B)** Wild type and *scat* cells were stained with Hoechst dye (10 μg/mL) and analyzed as individual erythropoietic populations by flow cytometry. **(C)** Representative cell cycle patterns are suggestive of an accumulation of cells in the G_0_/G_1_ phase during erythropoiesis in *scat* mice. Representative pattern from five independent experiments.

We previously reported an accumulation of erythroid precursors at the polychromatophilic and orthochromatic stages in the *scat* mouse model (Blanc et al., 2012), suggesting potential cell cycle defects. Therefore, we isolated the bone marrow from wild type and *scat* mice and measured the cell cycle progression at each of the erythroid differentiation stages using the DNA-binding dye Hoechst-33342 along with the erythroid cell surface markers CD44, Ter119, and FSC. Throughout differentiation, erythroid cells lose the adhesion molecule CD44, gain the membrane glycoprotein associated-Ter119, and decrease in size, allowing analysis of distinct developing populations (Liu et al., 2013; **Figure 1A**). While the pattern was similar between wild type and *scat* within the proerythroblast population, we observed different patterns of DNA content at the basophilic and polychromatophilic stages that demonstrate a failure to progress out of G_0_/G_1_ into S or G_2_ in *scat*, indicating a defect at the G_1_ checkpoint. However, we observed that the pattern either normalizes or becomes indiscernible by the orthochromatic stage, as orthochromatic erythroblasts in both *scat* and wild type mice are almost entirely in the G_0_/G_1_ phase, consistent with previously published studies (Pop et al., 2010; **Figures 1B,C**). Thus, we conclude that *Rasa3* plays a critical role in the G_1_ checkpoint at the basophilic and polychromatophilic stages of erythroid differentiation.

### Reactive Oxygen Species (ROS) Accumulate at the Proerythroblastic and Reticulocyte Stages in *scat*

**FIGURE 2:**
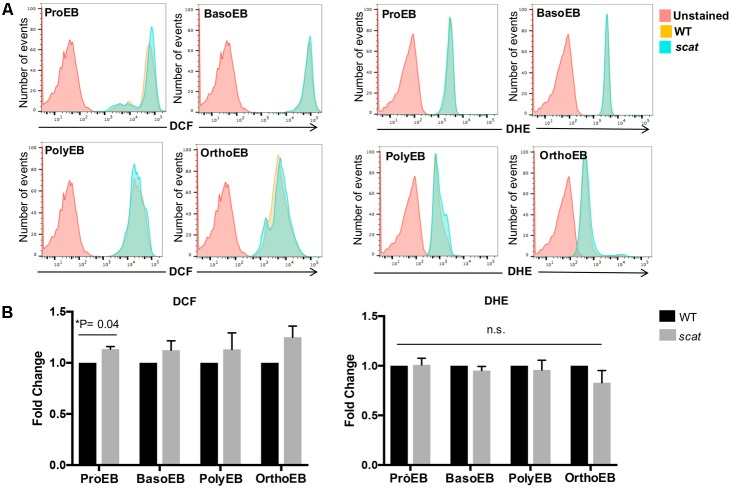
There is an increase in peroxide levels at the proerythroblastic stage of erythropoiesis in the spleen of *scat* mice. **(A)** Representative histograms showing the levels of DCF (four left panels) and DHE (four right panels) in the spleen measured as fluorescence intensity. **(B)** Quantification of DCF and DHE levels by population in the spleen of wild type and *scat* mice. Measured as fold change normalized to wild type (*n* = 6 for DCF and *n* = 14 for DHE).

Having shown that the cell cycle is altered specifically at the basophilic and polychromatophilic stages, we then hypothesized that increased levels of ROS could be involved in the mechanism leading to crisis in *scat*. To evaluate cytosolic peroxide and superoxide generation in wild type and *scat*, we used DCF and DHE, respectively, as previously described by George et al. (2013). We assessed ROS production in the spleen, bone marrow, and peripheral blood of the mice. A qualitative overlay of the superoxide and general oxidative stress signals in the spleens of wild type and *scat* indicate a relatively similar level of ROS levels (**Figure 2A**). However, when we quantified these ROS levels, we observed a significant increase in the peroxide levels at the proerythroblastic stage of *scat* spleen (**Figure 2B**; *P* < 0.05). We also noticed a trend toward increased levels of ROS throughout erythroid differentiation (**Figure 2B**, left panel). The same trend is observed in the bone marrow (Supplementary Figure 1). This increase in ROS does not seem to be due to an increase in cytosolic superoxide, as DHE levels remain unchanged in *scat* throughout differentiation, and comparable to those observed in WT (**Figure 2B**, right panel and Supplementary Figure 1).

**FIGURE 3:**
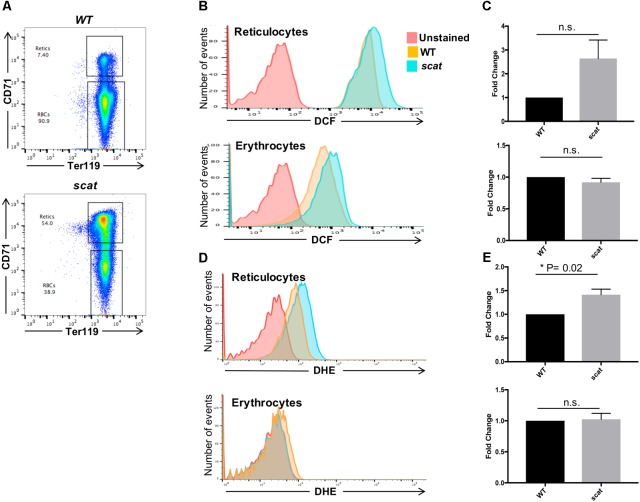
Increased ROS in *scat* crisis reticulocytes. **(A)** Representative gating of reticulocytes and erythrocytes based on levels of CD71 in wild type and *scat* peripheral blood. **(B)** Representative histograms showing the levels of DCF measured as fluorescence intensity in reticulocytes and erythrocytes. **(C)** Quantification of DCF levels in reticulocytes and erythrocytes based on fold change. **(D)** Representative histograms showing the levels of DHE measured as fluorescence intensity in reticulocytes and erythrocytes. **(E)** Quantification of DHE levels in reticulocytes and erythrocytes based on fold change (*n* = 11 for DCF and *n* = 9 for DHE).

Reactive oxygen species play a role in the regulation of the red cell lifespan (Friedman et al., 2004). Therefore, we evaluated these ROS levels in the peripheral blood of the mice. By using CD71 and Ter119 as markers, one can discriminate between reticulocytes and erythrocytes (**Figure 3A**). Due to the young age of the mice (less than 3 weeks old), there is a noticeable amount of reticulocytes in the bloodstream of WT animals. We observed that the trend toward increased DCF levels seen in the spleen (**Figure 2B**) is also present in *scat* reticulocytes (**Figures 3B,C**, upper panels). However, the increased levels of ROS normalize during reticulocyte maturation, as erythrocytes do not show an increase in DCF (**Figures 3B,C**, lower panels). Surprisingly, and unlike what we observed in the spleen, DHE levels are increased 1.4 times in *scat* reticulocytes compared to WT (**Figures 3D,E**, upper panels; *P* = 0.02). Nevertheless, this increase is transient, and the DHE levels normalize at the erythrocyte stage (**Figures 3D,E**, lower panels). Taken together, these results suggest that there is a transient increase in ROS, both early and late in erythroid differentiation in *scat*.

### Mitochondria Are Eliminated Normally at the Reticulocyte Stage in the *scat* Mice

**FIGURE 4:**
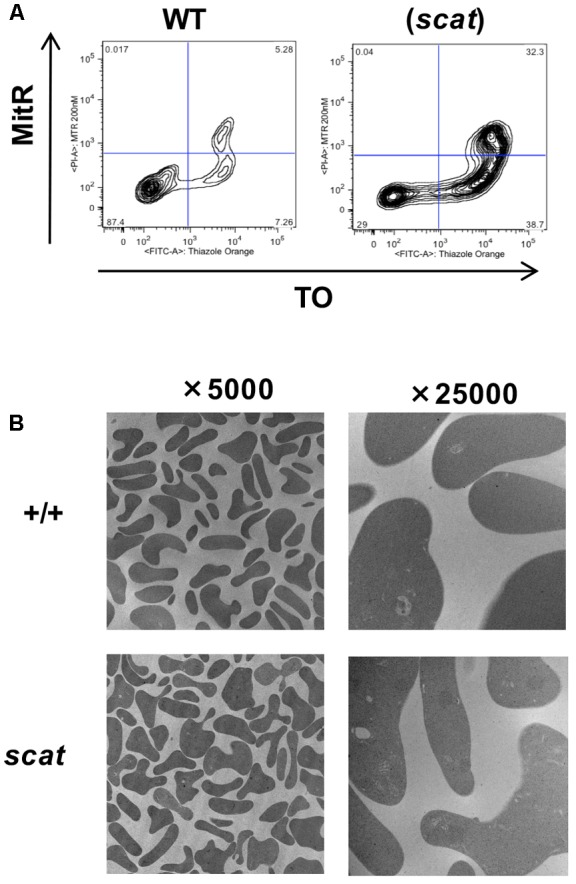
Mitochondria are removed normally during erythropoiesis in *scat* mice. **(A)** Analysis of wild type and *scat* peripheral blood by flow cytometry using markers for mitochondria (MitR) and RNA content of reticulocytes (TO). Early reticulocytes are ribosome-positive, mitochondria-positive (upper right quadrant), late reticulocytes ribosome-positive, mitochondria-negative (lower right quadrant), and retained mitochondria ribosome-negative, mitochondria-positive (upper left quadrant). No evidence for increased mitochondrial retention is seen. Representative pattern from five independent experiments. **(B)** Electron microscopy of RBCs in wild type and *scat* mice shows no evidence of mitochondria retention.

Mitochondria are an important source of ROS and are among the last organelles to be eliminated at the reticulocyte stage (Ney, 2011). Therefore, we assessed mitochondrial clearance during reticulocyte maturation in both WT and *scat* using MTR and TO as markers for mitochondria and reticulocyte maturation by RNA content, respectively. As shown in **Figure 4A**, no evidence of abnormal mitochondrial retention is seen in *scat.* While there is an increased overall number of reticulocytes in *scat* compared to WT due to the compensatory reticulocytosis, there are no TO^-^/MitR^+^ events, indicating no mature reticulocytes (TO^-^) with mitochondria present. In addition, electron microscopy performed on the peripheral blood of WT and *scat* animals demonstrates that no aberrant mitochondria are observed in the *scat* reticulocytes (**Figure 4B**). Thus, we conclude that the increased ROS levels observed at the reticulocyte stage are not a byproduct of improper mitochondria elimination.

### Apoptosis Is Decreased in *scat* During Erythropoiesis

**FIGURE 5:**
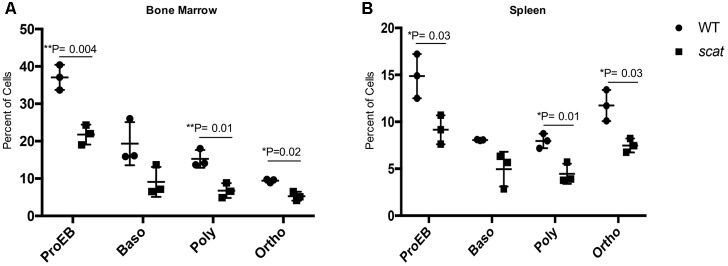
Early apoptosis is significantly decreased in *scat* mice at three distinct erythroid populations. **(A)** Quantification of early apoptosis in the bone marrow of wild type and *scat* mice at the proerythroblastic, basophilic, polychromatophilic, and orthochromatic erythroid differentiation stages. **(B)** Quantification of early apoptosis in the spleen of wild type and *scat* mice at the proerythroblastic, basophilic, polychromatophilic, and orthochromatic erythroid differentiation stages (*n* = 3).

Given the increased ROS in the reticulocytes and the overall cellular stress associated with the bone marrow failure observed in *scat*, we elected to investigate apoptosis at each stage of erythroid differentiation in the bone marrow and spleen. To do so, we used the markers Annexin V and 7AAD in addition to CD44, Ter119, and FSC as described previously (Liu et al., 2013). No significant increase in the overall percentage of early apoptotic (Annexin V^+^ 7AAD^-^), late apoptotic (Annexin V^+^ 7AAD^+^), or necrotic (Annexin V^-^ 7AAD^+^) cells in *scat* were seen compared to their littermate controls. However, we observed a significant decrease in the percentage of late apoptotic cells in the bone marrow (Supplementary Figure 2; *P* < 0.05). Additionally, when we quantified early apoptosis based on each individual erythroid population, we noticed a significant decrease in apoptosis in both scat bone marrow and spleen proerythroblastic, polychromatic, and orthochromatic stages (**Figures 5A,B**; *P* < 0.05). Together, these data suggest that loss of function in *Rasa3* leads to decreased apoptosis.

### *scat* Mice Demonstrate Decreased Plasma and Serum Levels of Galectin-1

**FIGURE 6:**
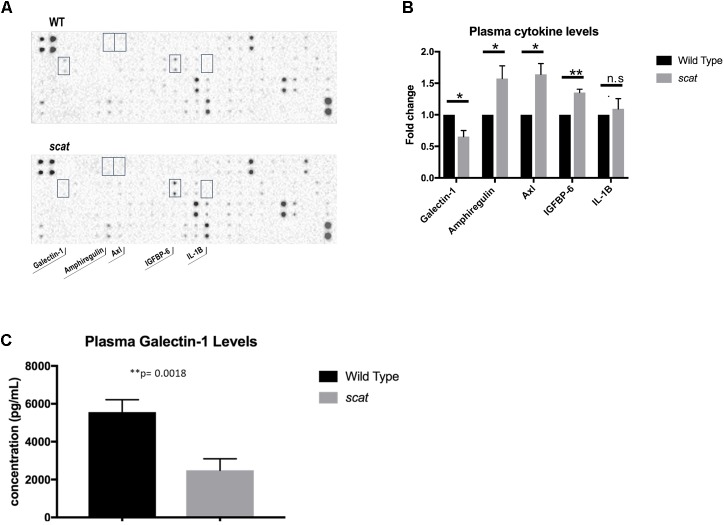
Levels of specific cytokines, including galectin-1, are altered in *scat* plasma and serum. **(A)** Analysis of plasma samples from *scat* and littermate WT mice by cytokine array reveals significant differences in four cytokines: galectin-1, amphiregulin, Axl, IGFBP-6, and no significant difference in levels of IL-1B (*n* = 3). **(B)** Quantification of the fold change of the cytokines specified in **A**; galectin-1: *p* = 0.0154, amphiregulin: *p* = 0.0366, Axl: *p* = 0.0135, IGFBP-6: *p* = 0.0011, IL-1B: *p* = 0.5825. **(C)** ELISA analysis of plasma and serum samples diluted 1:10 verifies significantly decreased levels of galectin-1 in *scat* samples compared to littermate WT (*n* = 15; *p* = 0.0018). ^∗^*p* < 0.05 and ^∗∗^*p* < 0.01.

One of the most striking aspects of the *scat* phenotype is the cyclic remission that includes normalization or near normalization of hematologic parameters and external physical manifestations of anemia and thrombocytopenia. The fact that remission occurs without a correction to RASA3’s aberrant location in the cytosol led to the hypothesis that a secreted factor may be mediating its onset. To test this hypothesis, we analyzed plasma cytokine profiles by cytokine array and identified several targets of interest, including galectin-1 (**Figure 6A**). Galectin-1 is a known mediator of stromal cell-to-stem cell interactions and transduction of intracellular signaling in the bone marrow hematopoietic niche (Liu and Rabinovich, 2010; Rabinovich and Vidal, 2011), and its levels were significantly decreased in *scat* mice during crisis compared to wild type (**Figure 6B**, 23326.5 ± 21439.72 vs. 31019.63897 ± 20110.65161; *P* < 0.05), making it our initial focus. Interestingly, well-known inflammatory mediators such as IL-1β and IL-6, were not significantly different between wild type and *scat*. To verify these results, we performed an ELISA on serum and plasma samples from *scat* and littermate wild type mice and indeed found significantly decreased levels of galectin-1 in samples from *scat* mice (**Figure 6C**, 2487.917 ± 606.1 pg/mL vs. 5562.417 ± 652.4 pg/mL; *P* < 0.05). Together, these results suggest that galectin-1 may play a role in the cyclic phenotype or serve as a biomarker of the crisis-remission transition observed in *scat*.

## Discussion

**FIGURE 7:**
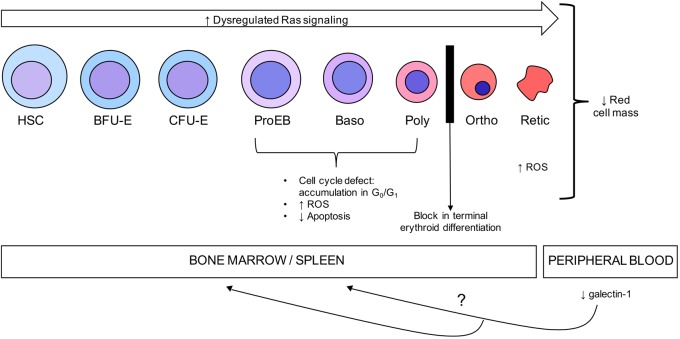
Model of the mechanisms of anemia in the *scat* mouse model. Mutant RASA3 is rendered non-functional by its mislocalization to the cytosol, leading to dysregulated Ras signaling throughout erythroid differentiation. This dysregulated signaling is associated with an accumulation in the G_0_/G_1_ phase of the cell cycle in early erythroid precursors, impairing proper proliferation and differentiation and contributing to decreased red cell mass, despite decreased apoptosis in these populations. A block in terminal differentiation with an accumulation at the polychromatic/orthochromatic stages, as well as increased ROS accumulation in the reticulocyte stage further contribute to the anemia seen as a result of the loss of functional RASA3. Secreted factors, such as galectin-1, may contribute to mediating remission in the erythropoietic microenvironment while RASA3 remains mislocalized.

In this study, we elucidate mechanisms contributing to anemia in *scat* mice during crisis events and propose a model in which loss of RASA3 function during erythropoiesis leads to dysregulation of several key cellular processes with the final result of anemia (**Figure 7**). In the bone marrow and spleen, impaired cell cycle progression and increased ROS inhibit proper proliferation and terminal differentiation, leading to eventual decreased red cell output, despite decreased apoptosis. In the periphery, increased ROS further contribute to anemia and secreted factors such as galectin-1 have a yet to be elucidated role in modulating erythropoiesis. The *scat* model is very unique in the context of existing mouse models of inherited bone marrow failures. Most current models focus on the immune-mediated pathogenesis of acquired AA (Scheinberg and Chen, 2013), and models of congenital AA have focused on mutations in DNA-repair pathways, such as the *Fanc^-/-^* models and more recent *Brca^-/-^* model (Parmar et al., 2009; Vasanthakumar et al., 2016). Therefore, *scat* and its loss of function mutation in *Rasa3* implicate a unique signaling axis in congenital AA. Further, though these other models of congenital AA have similar hematopoietic defects and possibly convergent molecular mechanisms, *scat* is the only existing model with a cyclic phenotype that offers key opportunities to study both the onset and remission of some aspects of bone marrow failure.

Cell cycle progression and erythropoiesis are intricately co-regulated. Proper alignment of cell cycle status with phases of erythroid maturation is crucial for regulating the transition from proliferative progenitors to increasingly differentiated precursors, allowing production of a sufficient number of fully mature red cells. Known cell cycle regulators have been shown to play key cell-intrinsic roles in promoting terminal erythroid differentiation (Sankaran et al., 2008). Further, Ras/Raf/MEK/ERK and Ras/PI3K/Akt pathways are known to control the transcription or regulation of key cell cycle regulators (Lee et al., 2008). Therefore, we examined the cell cycle status of individual erythroid differentiation populations in wild type and *scat* bone marrow. Our results demonstrate a pattern of accumulation in the G_0_/G_1_ phase in *scat*, suggestive of a defect at the G_1_ checkpoint that normalizes or becomes indiscernible by the orthochromatic stage. During normal erythropoiesis, most orthochromatic erythroblasts have exited the cell cycle and entered G_0_/G_1_. The G_1_ checkpoint integrates various intracellular signaling cascades, including pathways downstream of Ras, and either promotes or prevents DNA replication. Because loss of RASA3 function leads to increased active, GTP-bound Ras in *scat*, changes in Ras signaling pathways are likely influencing cell cycle progression. As mentioned above, the polychromatophilic to orthochromatic transition represents a key point of cell cycle exit during erythropoiesis that allows final terminal differentiation and enucleation. Prior work characterizing the erythroid differentiation defect in *scat* demonstrated an accumulation specifically at the polychromatophilic/orthochromatic stages. This accumulation therefore aligns with and supports the notion of aberrant cell cycling in *scat* erythropoiesis. Finally, many inherited BMFS have a predisposition for hematologic malignancies that are characterized by dysregulated cell cycling (Al-Rahawan et al., 2008). Therefore, the altered cell cycle profiles observed in *scat* further support its characterization as a model of inherited bone marrow failure. Future work will characterize exactly which cell cycle regulators are being affected and how altered Ras signaling mediates these changes. We will notably investigate the role played by cyclins and CDKs in the regulation of the cell cycle. Due to the fragile nature of the *scat* mice in crisis, we were unable to inject Bromodeoxyuridine (BrdU) that would have allowed quantification of individual G_0_/G_1_, S, and G_2_/M phases. However, even with our current methods, we were able to observe this consistent trend toward G_0_/G_1_ accumulation in *scat.*

We also identified increased ROS in *scat*, most notably the accumulation of superoxide in reticulocytes. ROS are known to negatively impact RBC health and lifespan, indicating that their increase likely contributes to the anemia of *scat* (Friedman et al., 2004). RASA3 is a known negative regulator of Ras activity, and previous studies have determined that upregulation of active Ras proteins can lead to increased intracellular ROS concentrations, further supporting a role for ROS in the *scat* phenotype (Zamkova et al., 2013). Impaired hemoglobin formation can also be a source of ROS. Accordingly, we previously documented hemoglobinization defects in *scat* (Blanc et al., 2012). Further studies will elucidate the mechanism(s) of increased ROS during erythropoiesis in *scat* by examining the activity of Ras isoforms, mitochondrial metabolism prior to proper removal, and hemoglobin synthesis. A surprising finding of the present work is the decrease in apoptosis seen in *scat* erythroid populations. Given the anemia and overall poor health of *scat*, one might expect to see an increase in apoptosis; however, this was not the case. Active H, K, and N-Ras are oncogenic proteins known to play a significant role in cell proliferation. Previous studies have shown that an overexpression of Ras isoforms leads to a block in terminal erythroid differentiation accompanied by increased proliferation of earlier erythroid populations (Zhang et al., 2003). Additionally, the same study demonstrated that the introduction of oncogenic Ras into erythroleukemia cells resulted in a block in terminal differentiation and extended proliferation with no effect on apoptosis (Zhang et al., 2003). This ability of precursor cells to survive and proliferate longer due to increased Ras signaling seen with loss of RASA3’s negative regulation could potentially explain why apoptosis is decreased in *scat* compared to wild type mice. The mechanism of the interesting cyclic phenotype of the *scat* mouse has yet to be elucidated and likely holds key insights into general mechanisms of onset and resolution of BMFS. We hypothesized that secreted factors may be playing a role in mediating remission and thus compared the plasma cytokine profiles of *scat* crisis and wild type mice. Galectin-1 was consistently decreased in *scat* compared to wild type, but levels of common inflammatory mediators such as IL-6 and IL-1β were unchanged. Galectins, a family of β-galactoside binding proteins, have been shown to play key roles in the bone marrow niche and the erythroblastic island, indicating that these lectins have the capacity to modulate the maturation of hematopoietic cells. Within the bone marrow niche, galectins are known mediators of stromal cell-hematopoietic cell interactions (Rabinovich and Vidal, 2011). Galectin-5, a member of the same subfamily as galectin-1, has been specifically implicated in protein sorting and regulation of exosomal uptake in rat erythroblastic islands (Barres et al., 2010). Further, galectins are also known modulators of various mitogen-activated protein kinase (MAPK) signaling pathways in several cell types, including key pathways (PI3K/Akt, Raf/Mek/MAPK) downstream of Ras that are likely to be altered with loss of RASA3 (Masamune et al., 2006; Shimizu et al., 2009). In this context, the ability of galectin-1 to modulate these signaling pathways is of great interest. Though existing data is intriguing and suggestive of galectin-1’s importance, future studies will be required to prove any mechanistic role for galectin-1 in the cyclic bone marrow failure phenotype, rather than its decrease simply being an effect of loss of RASA3 function across all cell types in the mouse, or a generic serum biomarker of disease.

## Author Contributions

EH and EB designed and performed the research, analyzed the data, and wrote the manuscript. JP, SC, and YZ performed the research, analyzed the data, and edited the manuscript. LP and LB designed the research, analyzed the data, and wrote the manuscript.

## Conflict of Interest Statement

The authors declare that the research was conducted in the absence of any commercial or financial relationships that could be construed as a potential conflict of interest.
