# Supplementary figures and images for: Increased Reactive Oxygen Species and Cell Cycle Defects Contribute to Anemia in the RASA3 Mutant Mouse Model scat
Source: Front Physiol. 2018 Jun 5;9:689. doi: 10.3389/fphys.2018.00689 (PMC5996270; doi:10.3389/fphys.2018.00689)

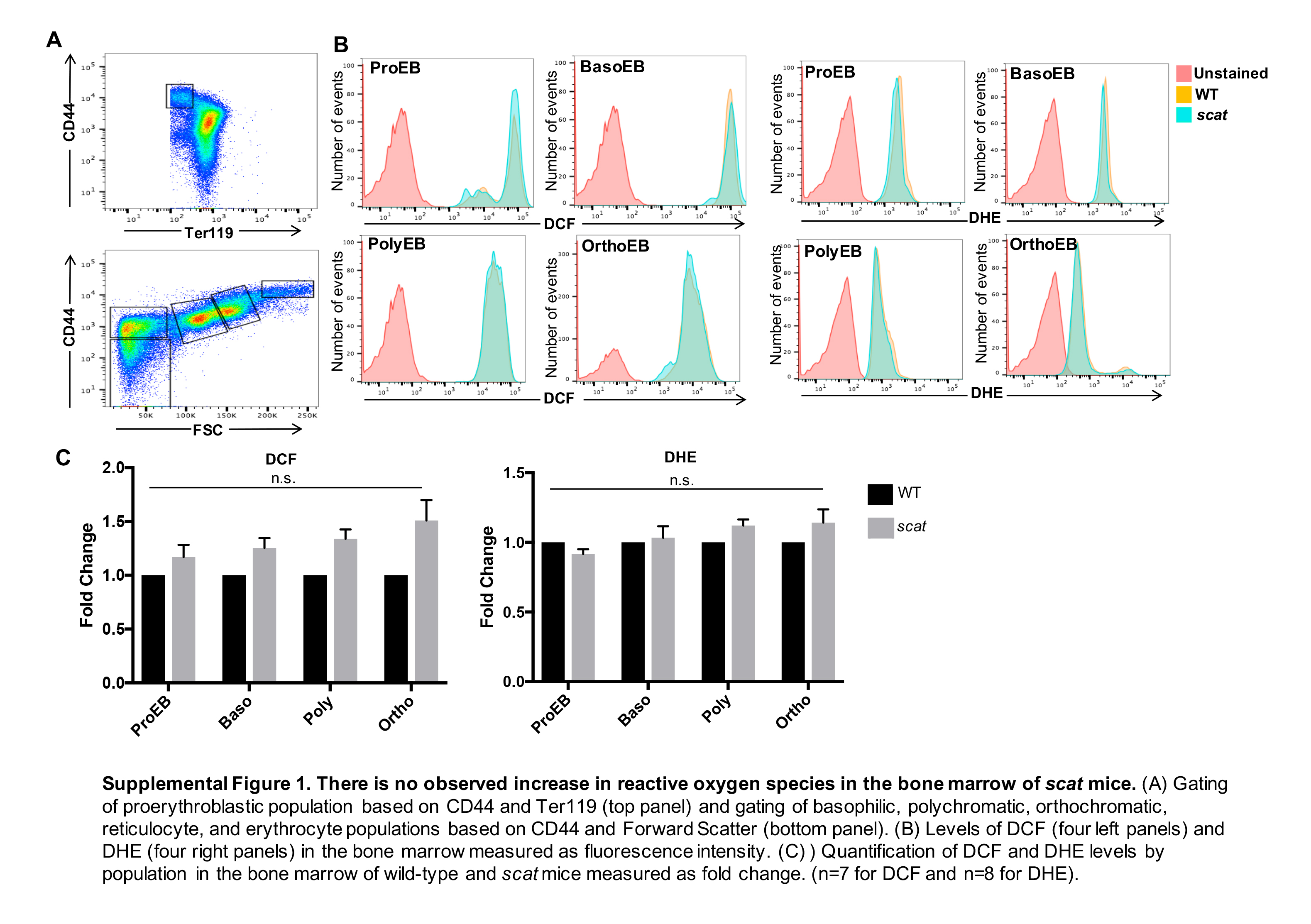

Supplement: Supplementary file 1 [file Image_1.JPEG]

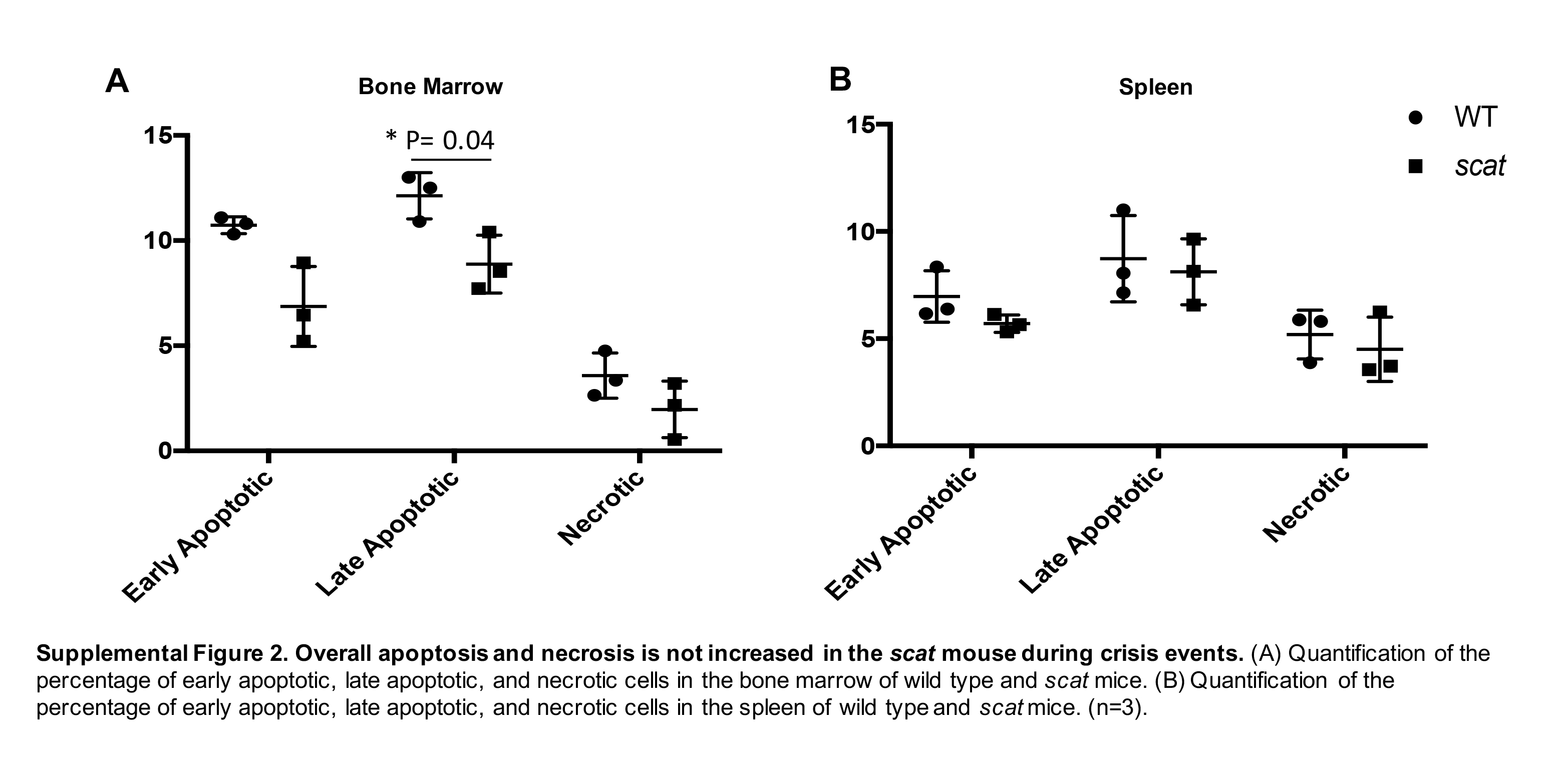

Supplement: Supplementary file 2 [file Image_2.JPEG]
